# Physically Reshaped Silver Microplates Formed Monolayer Assemblies at Air/Water Interface as High-Performance SERS Substrates

**DOI:** 10.3390/s26061943

**Published:** 2026-03-19

**Authors:** Aoran Cui, Shaojing Su, Tianle Wang, Yaqin Liao, Shikuan Yang

**Affiliations:** 1 School of Materials Science and Engineering, Zhejiang University, Hangzhou 310027, China; 12126053@zju.edu.cn (A.C.); 3220102751@zju.edu.cn (T.W.); 2 Dongfang Electric Corporation Academy of Science and Technology, Co., Ltd., Chengdu 610093, China; susj@dongfang.com

**Keywords:** silver microplates, interfacial self-assembly, SERS substrates

## Abstract

Surface-enhanced Raman scattering (SERS) holds great promise for ultrasensitive chemical analysis but is often limited by the trade-off between performance and fabrication simplicity. This work presents a facile strategy to prepare monolayer silver microplates combining the top-down and bottom-up fabrication concepts. Silver microplates with uniform nanoscale thickness (~93.5 nm) and micron-scale lateral size (D_50_ = 3.33 µm) are prepared via a scalable mechanical ball-milling process. These silver microplates served as building blocks for spontaneous interfacial self-assembly at the air/water interface to form a macroscopically continuous monolayer film. The silver microplate monolayer film is transferred onto a plasma-treated silicon wafer as a SERS substrate. The resulting SERS substrate exhibits a porous, network-like microstructure composed of densely packed microplates, which generates a high density of electromagnetic hot spots at the nanogaps. Using Rhodamine 6G as a probe molecule, the substrate demonstrates a SERS detection limit of as low as 1 nM and good spatial uniformity with a relative standard deviation of ~9.94%. This study provides a cost-effective and scalable self-assembly route of physically reshaped silver microplates to fabricate high-performance SERS substrates.

## 1. Introduction

Surface-enhanced Raman scattering (SERS) has established itself as an exceptionally powerful analytical technique, capable of providing fingerprint-like vibrational spectra of molecules at ultralow concentrations, even down to the single-molecule level [1,2]. This remarkable sensitivity stems from the enormous amplification of the local electromagnetic field generated by the excitation of localized surface plasmon resonances (LSPRs) in metallic nanostructures, typically silver or gold [3,4]. Consequently, the performance of a SERS substrate is intrinsically linked to the architecture of its constituent nanostructures, which govern the density, intensity, and uniformity of these plasmonic “hot spots” [5,6]. Over the past decades, significant efforts have been devoted to fabricating SERS-active substrates. These can be broadly categorized into two paradigms: top-down lithography and bottom-up chemical assembly [7]. Lithographic techniques, such as electron-beam lithography and nanoimprinting, offer exquisite control over nanostructure geometry and arrangement, enabling the rational design of hot spots and excellent reproducibility [8]. However, their high cost, limited throughput, and constraints on substrate size and material choice often hinder widespread applications [9]. On the other hand, bottom-up approaches, particularly the assembly of colloidal nanoparticles (NPs) through drop-casting, spin-coating, or Langmuir–Blodgett techniques, provide a more scalable and cost-effective alternative route [10]. These methods frequently suffer from challenges related to the uncontrolled aggregation of NPs, leading to poor spatial uniformity, limited hot-spot density, and weak mechanical adhesion of the active layer to the substrate [11]. Therefore, a persistent challenge in the field remains the development of a simple, scalable, and low-cost fabrication strategy that can fabricate high SERS performance.

In this context, the self-assembly of two-dimensional (2D) metallic nanostructures, such as silver microplates or nanosheets, at interfaces presents a promising avenue [12]. Compared to spherical NPs, microplates exhibit stronger and more tunable plasmonic resonances and possess high surface area [13]. Their anisotropic shape favors face-to-face stacking and lateral organization, which can lead to the formation of continuous, porous films with a high density of inter-plate nanogaps acting as ideal configurations for generating intense and homogeneous electromagnetic enhancement sites [14]. However, the controlled synthesis of such microplates with uniform thickness and their subsequent organization into large-area, robust films remain a challenge [15]. Conventional wet-chemical syntheses of microplates often involve complex protocols, capping agents, and poor control over thickness distributions [16].

In this study, we present a facile approach for fabricating high-performance SERS substrates employing a scalable top-down synthesis of silver microplates with a spontaneous air/water interfacial self-assembly process [17]. Unlike conventional methods, our approach integrates the top-down mechanical reshaping of spherical silver microparticles into microplates with the bottom-up interfacial assembly process, enabling the production of large-area silver microplate monolayer films with high-density plasmonic hot spots. First, silver microplate powder with micrometer lateral dimensions and a uniform thickness was prepared via a straightforward mechanical ball-milling and drying process. These microplates were subsequently used as structural building blocks to spontaneously assemble into a continuous film at the air–liquid interface. The microplate film was transferred onto a plasma-treated silicon wafer to form a stably adhered SERS substrate [18]. We systematically investigated the synthesis and characterization of the microplates, the self-assembly mechanism and the microplate film morphology, as well as the evaluation of the SERS performance. The results demonstrate that the obtained SERS substrate exhibits a detection limit of as low as 1 nM for Rhodamine 6G with good signal uniformity (RSD ≈ 9.94%), benefiting from the high density of hot spots formed within the porous network microstructure of the microplate film. This work not only demonstrates a high-performance SERS substrate fabricated via a simple and low-cost route, but also elucidates the relationship between the mesoscale assembly morphology and the macroscopic functional performance, providing guidance for the future design of plasmonic metasurfaces and sensing platforms [19].

## 2. Materials and Methods

### 2.1. Materials

Silver micro-powder (spherical particles, 99.9% purity) was used as the precursor material. Zirconia grinding beads with diameters of 1 mm and 7 mm were employed as the milling media. The grinding aid (denoted as G1, oleic acid) (Sinopharm Chemical Reagent Co., Ltd., Shanghai, China) and the surface modifier (denoted as G2, stearic acid) (Sinopharm Chemical Reagent Co., Ltd., Shanghai, China) were used as received without further purification. Ethanol (analytical grade) (Sinopharm Chemical Reagent Co., Ltd., Shanghai, China) and deionized water (DI water, resistivity > 18.2 MΩ·cm) were mixed to prepare the solvent system.

### 2.2. Characterizations

Scanning electron microscopy (SEM) images of silver microflake powder and silver microplate monolayer films were carried out on a Hitachi SU-8010 scanning microscope (Hitachi, Tokyo, Japan). The photographs were taken by a Panasonic DC-S5 digital camera (Panasonic, Osaka, Japan). The SERS measurement was conducted on a confocal Raman microscopic system (LabRAM HR Evolution, Horiba, Villebon-sur-Yvette, France) equipped with a 532 nm laser operating at a power of about 0.25 mW. The accumulation time for the SERS signals was 1 s. At least 15 spots on the same SERS substrate were examined. The spectra were averaged for final analysis.

### 2.3. Synthesis of Silver Microflake Powder

Silver microparticles were used as the precursor material. The spherical silver microparticles were synthesized via a seed-mediated growth method [20]. First, silver nanoseeds (50–100 nm) were prepared by rapidly mixing AgNO_3_ and ascorbic acid into a polyvinyl pyrrolidone (PVP) (K90) solution at 50 °C. These seeds were then used to induce the growth of micrometer-sized silver spheres in a reaction system containing PVP (K60), AgNO_3_, and ascorbic acid at 30 °C. The key to preparing uniform spherical particles lies in controlling the competition between the nucleation and the growth kinetics. At a high saturation status, burst nucleation occurs to yield nanoparticles after properly prohibiting the aggregation. In contrast, under a low saturation condition with a low reaction rate, the seeds act as growth sites to guide the growth of uniform microparticles [20].

The silver microflake powder was synthesized via a wet mechanical ball-milling process using spherical silver microparticles as the precursor. Zirconia grinding beads with diameters of 1 mm and 7 mm were employed as the milling media at a fixed bead-to-precursor weight ratio of 10:1. The milling was conducted in an ethanol–water mixture (60–80 vol% ethanol) medium, which was added to submerge the grinding beads and the silver microparticle powder. Low-speed stirring for 5 min was performed to ensure initial homogeneity. Subsequently, the grinding aid (G1, oleic acid) was added, followed by medium-speed mixing for 5 min before the surface modifier (G2, stearic acid) was introduced. The primary milling stage was then carried out at a high speed for 4 to 10 h to flatten the silver particles into a flake morphology.

Following the milling process, the resulting slurry was separated from the grinding beads using a sieve. The collected silver microflake slurry or “silver mud” was washed with deionized water for multiple cycles of centrifugation and re-dispersion processes. The microflakes achieved a stable, fly ash-like dispersion state and the electrical conductivity of the filtrate fell below 20 μS/cm after complete removal of the ionic impurities and processing additives. The purified silver microflakes were then transferred to trays and dehydrated in a forced-air circulation oven at 60 °C until a constant weight was achieved. The moisture content was less than 0.05 wt% in the final powder sample.

The dried silver microflake was gently de-agglomerated and passed through a 500-mesh sieve (aperture ~25 μm) to remove large aggregates, yielding the final free-flowing target silver microflake powder for subsequent self-assembly and characterization.

### 2.4. Fabrication of Self-Assembled Silver Microflake Films

Silver microflake film was assembled from the silver microplates prepared as described above. First, 0.05 g of dried silver microplates were dispersed in a 30 mL beaker containing a mixture of ethanol and water with a volume ratio of 3:2. We tested ethanol concentrations from 30% to 90%. The nanoplates exhibited optimal dispersion stability and interfacial film formation without aggregation or sinking at the concentration of 60%. Low ethanol concentrations led to aggregation and sedimentation, while high concentrations caused rapid evaporation and film rupture. Second, the silver microplate dispersions were kept undisturbed to allow them to assemble at the air–water interface. After 5 min, the silver microplates spontaneously assembled into a macroscopic continuous film at the air–liquid interface driven by the minimization of interfacial energy. Third, a piece of plasma-treated hydrophilic silicon water was used to pick up the assembled film from the bottom. In detail, the silicon substrate was treated with oxygen plasma at 50 W for 5 min under a pressure of 0.4 mbar to enhance hydrophilicity. The floating film was transferred by slowly dipping the substrate into the solution and withdrawing it at a 45° angle, ensuring conformal contact. A shiny silver microflake film was obtained after the water evaporated. This plasma treatment enhanced the hydrophilicity of the silicon substrate to make the silver microflake film strongly adhere to the substrate. Finally, a uniform and shiny silver microflake film was obtained.

## 3. Results and Discussion

### 3.1. Synthesis and Characterization of the Silver Microplates

**Figure 1:**
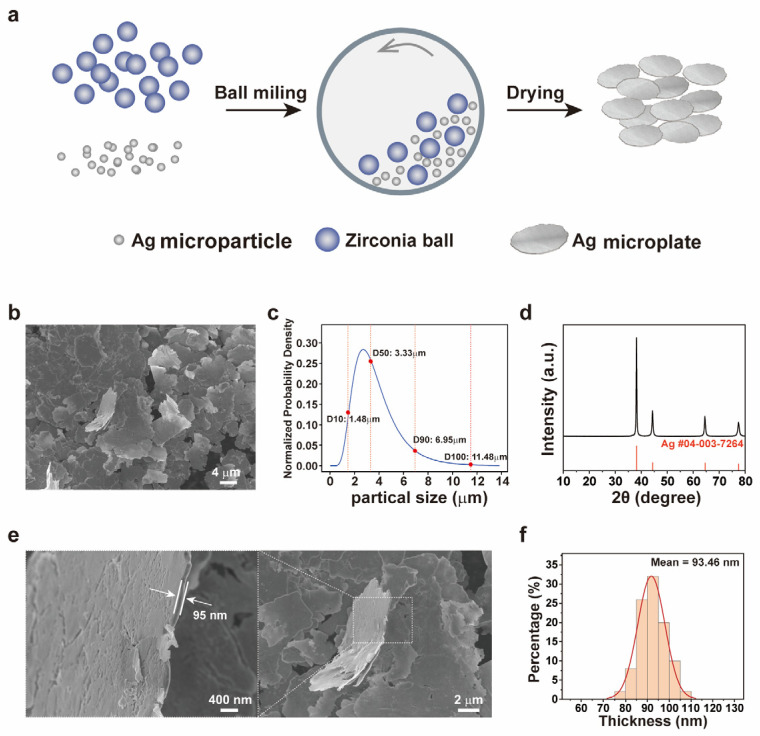
**Synthesis and characterization of silver microplates.** (**a**) Schematic of the mechanical ball-milling synthesis process of the silver microplates; (**b**) SEM image of the obtained silver microplates; (**c**) size distribution of the silver microplates, showing a median particle size (D_50_) of 3.33 μm; (**d**) XRD pattern of the silver microplates; (**e**) SEM images of the silver microplates at different magnifications; (**f**) thickness distribution of the silver microplates with an average thickness of about 93.5 nm.

Figure 1 systematically characterized the microstructure and the size distribution of the silver microplates prepared via the ball-milling method. Figure 1a illustrates the synthesis process. Silver microspheres and zirconia particles were uniformly mixed with a mechanical ball-milling machine. The silver microparticles (Figure S1, Supporting Information) had an average diameter of 1–3 μm. During the ball-milling process, these microparticles suffered from plastic deformation due to repeated collision processes and shear forces, gradually flattening then into plate-like structures. After cleaning and drying, the silver microplate powder was obtained. Figure 1b presents the scanning electron microscopy (SEM) image of the obtained silver microflake powder. The size analysis results (Figure 1c) indicated that the median particle size (D_50_) of the silver platelets was 3.33 μm with D10 and D_90_ values of 1.48 μm and 6.95 μm, respectively. The maximum size reached approximately 11.48 μm. This wide-range size distribution facilitated the formation of dense packing during subsequent self-assembly stages. The X-ray diffraction (XRD) pattern (Figure 1d) confirmed the crystalline phase of the material. All diffraction peaks corresponded to the standard peaks of silver (PDF#04-003-7264), indicating that the silver microplates were crystalline. Scanning electron microscopy (SEM) observations at different magnifications (Figure 1e) revealed the formation of uniformly thick platelet structures.

The thickness of the silver microplates was statistically evaluated by measuring more than 50 individual microplates (Figure 1f). The results indicated that the thickness of the microplates primarily ranged from 60 nm to 130 nm, with an average thickness of approximately 93.5 nm and with a relatively narrow size distribution. This uniform nanoscale thickness is crucial for their optical, electrical, and catalytic properties, because the thickness of the platelet structure directly influences its surface plasmon resonance effects and active specific surface area [21].

### 3.2. Self-Assembled Silver Microflake Films

**Figure 2:**
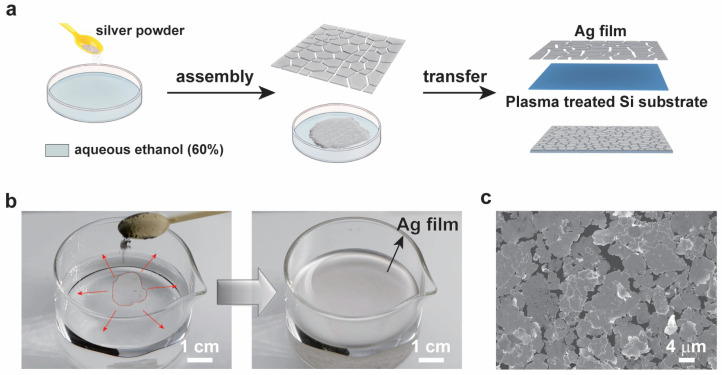
**Fabrication and structural analysis of the self-assembled silver microflake film.** (**a**) Schematic of the three-step process for interfacial self-assembly and silver microflake film transfer; (**b**) photograph of the macroscopically continuous film formed by silver microplates at the air–water interface; (**c**) SEM image of the silver microflake film transferred onto a piece of silicon substrate.

Leveraging the synthesized silver microplates as primary building blocks, large-area self-assembled silver microflake films were fabricated using an air–water interfacial self-assembly process [18]. The preparation procedure involves three steps (Figure 2a). First, the dry silver microplates were re-dispersed in a mixture of water and ethanol at a volume ratio of 3:2. The choice of this mixture solvent is critical for the self-assembly process. Ethanol reduces the surface tension of water, facilitating the adsorption and spreading of the hydrophobic silver microplates at the air–water interface [22]. Simultaneously, the water component provides sufficient polarity to stabilize the dispersion and prevent aggregation and sedimentation of the silver microflakes. This optimized solvent medium is crucial for controlling the surface energy and improving the stability of the microflakes during the subsequent assembly stage.

In the second step, the dispersion was left undisturbed, allowing the silver microplates to spontaneously migrate to and assemble at the air–liquid interface. This process is driven by the minimization of the overall free energy of the system, gearing the orientation adjustment of the microplates to reduce the contact area between their hydrophobic surfaces and the polar solvent. The microplates gradually organized into a continuous macroscopic film covering the interface, as shown in Figure 2b. The photograph and the video (Video S1, Supporting Information) clearly show a continuous reflective layer covering the entire air–water interface, indicating a successfully assembled large-area film. This interfacial self-assembly is a powerful bottom-up strategy for creating large-area monolayers or few-layer films of micro/nanoscale building blocks.

The final step was to transfer the floating microplate film onto a solid substrate. Here, a piece of hydrophilic silicon wafer treated by oxygen plasma was used. The oxygen plasma treatment thoroughly cleaned the surface and removed the organic contaminants, and, more importantly, generated a high density of hydrophilic silanol (-Si-OH) groups on the silicon surface. This dramatically enhanced the surface energy and improved the wettability of the substrate. The water contact angle decreased from 59° to 10° after oxygen plasma treatment (Figure S2, Supporting Information), allowing the silver microflake film to strongly adhere to the substrate. The silicon substrate was immersed in the water beneath the floating film. Slowly pulling out the substrate enabled the transfer of the silver microflake film onto the substrate. The strong hydrophilic interaction between the water layer adhering to the plasma-treated surface and the silver microflake film enabled a conformal transfer, resulting in significantly improved adhesion between the silver microflake film and the substrate.

The microstructure of the transferred film was examined by SEM (Figure 2c). The low-magnification image showed a continuous film over a large area. The high-magnification inset revealed that the porous, network-like film was composed of closely packed, overlapping silver microplates. The pores or interstices between the microplates were in the order of tens to hundreds of nanometers. This specific morphology is highly required for surface-enhanced Raman scattering (SERS) applications [23]. The dense packing ensures a high density of electromagnetic “hot spots”—regions of intense localized field enhancement typically existing in nanoscale gaps and at sharp edges between silver nanostructures. Furthermore, the porous characteristics allow analyte molecules to readily penetrate the film and access these hot-spot regions. Three-dimensional interference microscope analysis of the transferred silver microflake film revealed a surface roughness (Ra) of approximately 123 nm (Figure S3, Supporting Information), indicating a relatively textured surface preferred by SERS applications. This unique microstructure equipped within the interfacial self-assembly of pre-synthesized microplates provides the essential structural foundation for the plasmonic and SERS sensing applications.

### 3.3. Evaluation of the SERS Performance

We evaluated the SERS performance of the fabricated silver microflake film regarding sensitivity, uniformity, and stability. Rhodamine 6G (R6G) was used as a standard probe molecule. Different concentrations of R6G were detected using the silver microflake films.

**Figure 3:**
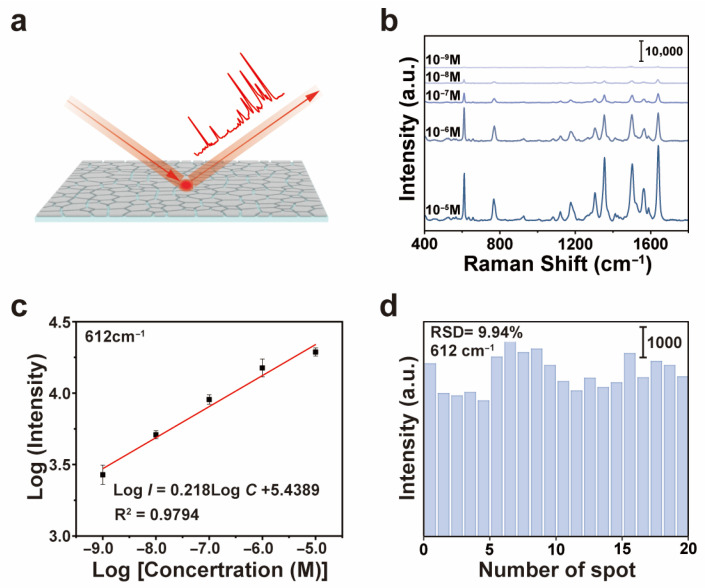
**Evaluation of SERS performance.** (**a**) Schematic of Raman characterization on the assembled silver film; (**b**) SERS spectra of Rhodamine 6G (R6G) on the silver film at different concentrations; (**c**) logarithmic plot of SERS intensity at 612 cm^−1^ versus R6G concentration, showing a linear range from 10^−5^ to 10^−9^ M; (**d**) RSD of the peak intensity at 612 cm^−1^ measured at 20 random locations.

The SERS performance is summarized in Figure 3. Characteristic Raman bands of R6G were clearly visible across a wide concentration range (Figure 3b). For quantitative analysis, the intensity variation in the SERS peak at 612 cm^−1^ for different concentrations of R6G aqueous solutions was monitored. This band is primarily attributed to the in-plane bending vibration of the xanthene ring skeleton in R6G, specifically involving the in-plane deformation of C–C–C bonds [24,25]. As shown in Figure 3c, this characteristic peak remained distinctly observable even at an ultralow R6G concentration of 1 × 10^−9^ M (1 nM). Even at 10^−9^ M, the peak at 612 cm^−1^ was still observable after baseline correction, as shown in the enlarged image (Figure S4, Supporting Information). The SERS performance of the self-assembled silver microflake film was on a par with or better than previously reported SERS substrates fabricated by more complex lithographic or chemical methods [26]. A linear relationship was built between the logarithmic concentration of R6G and the SERS signal intensity (at 612 cm^−1^) across four orders of magnitude (from 10^−5^ M to 10^−9^ M), indicating the possibility of quantitative analysis (Figure 3b). The limit of quantification (LOQ) was calculated to be 1.6 nM [27].

To evaluate the SERS detection reliability on the silver microflake film, SERS spectra were collected from 20 randomly selected spots on the SERS substrate using a fixed concentration of R6G (10^−5^ M). The results are plotted in Figure 3d. The relative standard deviation (RSD) of the peak intensity at 612 cm^−1^ over these 20 random spots was calculated to be 9.94%. This RSD value indicates good signal reproducibility and spatial uniformity over the SERS substrate. The SERS mapping over a 40 × 40 μm^2^ area (Figure S5, Supporting Information) suggested a dense distribution of hot spots across the silver microflake SERS substrate. The observed uniformity of the SERS signals can be attributed to the homogeneous microstructure of the self-assembled silver microflake film, owing to the relatively even distribution of the hot spots over macroscopic areas. The SERS enhancement factor (EF) was calculated using the standard formula EF = (I_SERS_/I_Raman_) × (N_Raman_/N_SERS_) [28]. For the 612 cm^−1^ SERS peak, the EF was estimated to be approximately 2.1 × 10^6^, indicating strong electromagnetic enhancement. The long-term stability of the SERS substrate was evaluated by measuring the SERS intensity after storage in ambient conditions for up to four weeks. The SERS substrate retained over 80% of its initial intensity at 612 cm^−1^ (Figure S6, Supporting Information), demonstrating good stability. To demonstrate the real application potential of the SERS substrate, SERS detection of tetramethylthiuram disulfide (TMTD), which is usually used as a pesticide, was performed (Figure S7, Supporting Information). Observable SERS peaks of TMTD were observed from a concentration of 0.1 nM, indicating the application potential of the silver microflake film in trace pesticide detection.

We compared the SERS performance of the silver microflake film with several recently reported SERS substrates fabricated by different methods. As summarized in Table 1, the silver microflake SERS substrate prepared by a simple, low-cost, and high-throughput fabrication method exhibited a detection limit of 1 nM for R6G, which was comparable to or even better than that of the previously reported SERS substrates [29,30,31,32].

The outstanding SERS performance can be correlated with the structural characteristics exhibited in Figure 1 and Figure 2. First, the uniform nanoscale thickness (~93 nm) of the silver microplates optimized their individual plasmonic response [33]. Second, the micrometer-scale lateral size and polydisperse distribution enable the formation of a densely packed continuous film with a high surface area [34]. More importantly, the self-assembly process creates a pervasive network of nanoscale gaps and junctions between adjacent and overlapping silver microplates. These junctions act as intense electromagnetic hot spots, providing the massive SERS signal enhancement required for single-molecule-level sensitivity [35]. The combination of the optimized building-block geometry and the dense packing creates abundant hot spots within the silver microflake film, resulting in superior sensitivity and uniformity.

## 4. Conclusions

In summary, we demonstrate a scalable and cost-effective method to fabricate high-performance SERS substrates through interfacial self-assembly of silver microplates at the air–water interface. Micrometer-scale, ultrathin silver microplates were synthesized by a physically reshaping ball-milling method. The silver microplates were spontaneously assembled at the air–water interface to form a dense film. The freestanding silver microflake film was transferred onto a piece of silicon substrate. The transferred film exhibited a porous microstructure composed of densely packed silver microplates, generating abundant electromagnetic hot spots at nanoscale gaps. The silver microplate film demonstrated high SERS sensitivity and high spatial uniformity. This approach effectively bridges nanomaterial synthesis and assembly to form functional membranes. The conductive silver microflake film holds great promise for applications in flexible electronics, catalysis, and tactile sensors, providing a foundation for constructing functional devices via interfacial self-assembly of nanoscale building units.

## Figures and Tables

**Table 1:** Comparison of the SERS performance of the silver microflake SERS substrate with that of the recently reported SERS substrates.

Substrate Type	Fabrication Method	LOD (R6G)	Fabrication Complexity	Cost	Reference
Ag nanoplate monolayer	Ball-milling + interfacial assembly	1 nM	Low	Low	This work
PLA/Ag NPs nanofiber	Electrospinning + plasma	0.1 nM	Moderate	Low	[29]
Ag NCs@PDMS	Interfacial assembly + polymer curing	1 nM	Moderate	Low	[30]
Ag@PDMS nanocavity	Bio-template stripping + sputtering	0.01 nM	Moderate	Moderate	[31]
Ag NCs@SiO_2_	Liquid–liquid interface transfer	1 pM	Moderate	Low	[32]

## Data Availability

Data are contained within the article.

## Supplementary Materials

The following supporting information can be downloaded at: https://www.mdpi.com/article/10.3390/s26061943/s1, Figure S1: SEM images of the spherical silver microparticle precursor used for the fabrication of silver plates; Figure S2: Changes in the water contact angles of the silicon wafer before and after plasma treatment: (a) Water contact angle of the silicon wafer before plasma treatment. (b) Water contact angle of the silicon wafer after plasma treatment; Figure S3: Micromorphology of the assembled silver film (area size: 15 μm × 15 μm). (a) 2D plan view from 3D interference microscope. (b) 3D stereoscopic image from 3D interference microscope; Figure S4: Raman spectrum of R6G at a concentration of 10^−9^ M on the silver film. The characteristic peak at 612 cm^−1^ is marked; Figure S5: SERS mapping of the 612 cm^−1^ peak of R6G molecules on the silver film over a 40 × 40 μm^2^ area; Figure S6: Long-term stability evaluation of the silver film substrate. The SERS intensity was measured before and after one month of storage under ambient conditions; Figure S7: SERS spectrum of Tetramethylthiuram Disulfide (TMTD) acquired on the silver film substrate; Video S1: Ag flakes.
