# Supplementary material for: Physically Reshaped Silver Microplates Formed Monolayer Assemblies at Air/Water Interface as High-Performance SERS Substrates
Source: Sensors (Basel). 2026 Mar 19;26(6):1943. doi: 10.3390/s26061943 (PMC13030420; doi:10.3390/s26061943)
Supplement: Supplementary file 1 [file sensors-26-01943-s001.zip › Supporting Information-revised version.pdf]

Supplementary information

Physically Reshaped Silver Microplates Formed Monolayer Assemblies at Air/Water Interface as High-Performance SERS Substrates

Aoran Cui 1,†, Shaojing Su 2,†, Tianle Wang 1, Yaqin Liao 2,* and Shikuan Yang 1,*

1 School of Materials Science and Engineering, Zhejiang University, Hangzhou 310027, China; 12126053@zju.edu.cn (A.C.); 3220102751@zju.edu.cn (T.W.)

2 Dongfang Electric Corporation Academy of Science and Technology, Co., Ltd., Chengdu 610093, China; susj@dongfang.com

* Correspondence: liaoyq@dongfang.com (Y.L.); shkyang@zju.edu.cn (S.Y.)

† These authors contributed equally to this work.

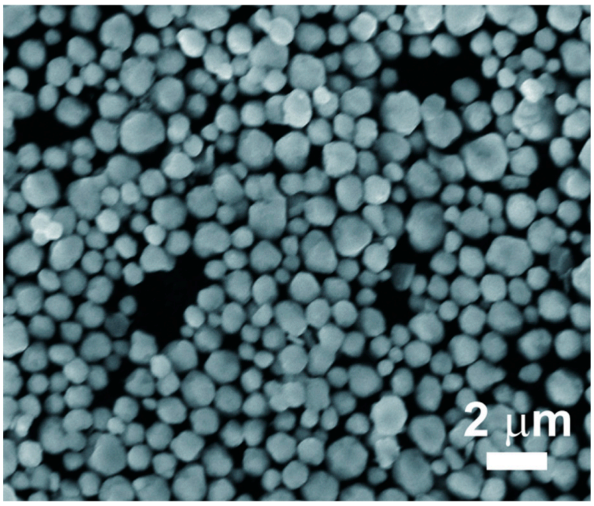

Figure S1. SEM images of the spherical silver microparticle precursor used for the fabrication of silver plates.

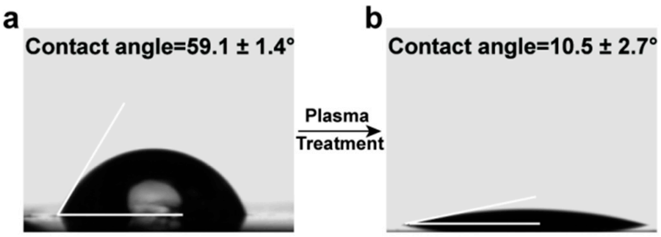

Figure S2. Changes in the water contact angles of the silicon wafer before and after plasma treatment. (a) Water contact angle of the silicon wafer before plasma treatment. (b) Water contact angle of the silicon wafer after plasma treatment.

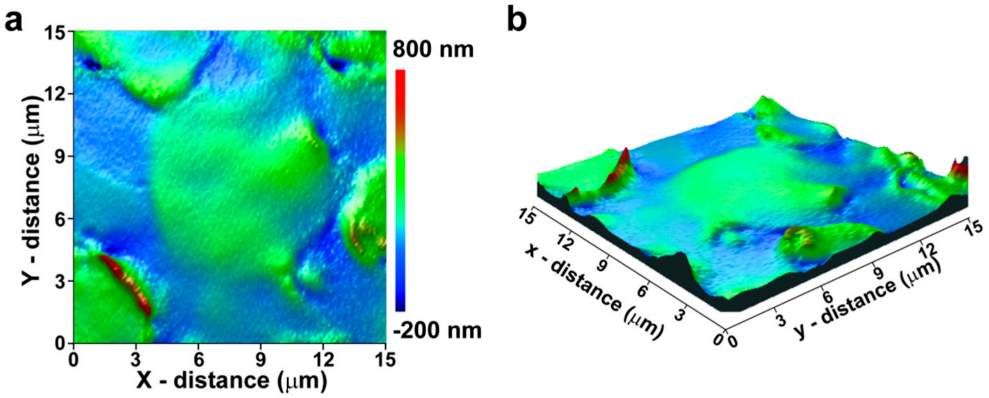

Figure S3. Micromorphology of the assembled silver film (area size: 15 μm × 15 μm). (a) 2D plan view from 3D interference microscope. (b) 3D stereoscopic image from 3D interference microscope.

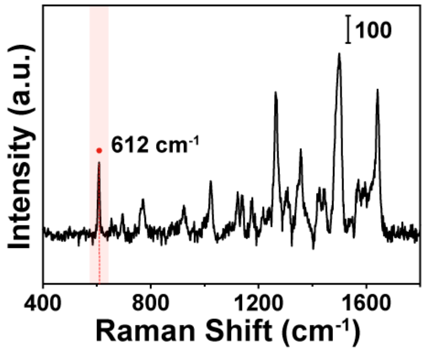

Figure S4. Raman spectrum of R6G at a concentration of 10^{-9} M on the silver film. The characteristic peak at 612 cm^{-1} is marked.

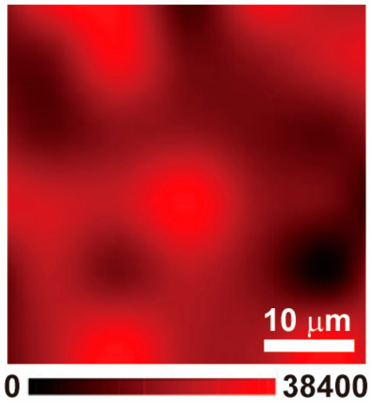

Figure S5. SERS mapping of the 612 cm^{-1} peak of R6G molecules on the silver film over a 40 × 40 $μm^2$ area.

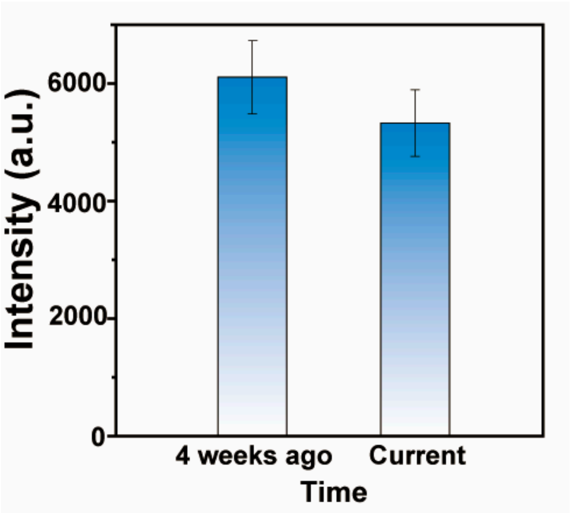

Figure S6. Long-term stability evaluation of the silver film substrate. The SERS intensity was measured before and after one month of storage under ambient conditions.

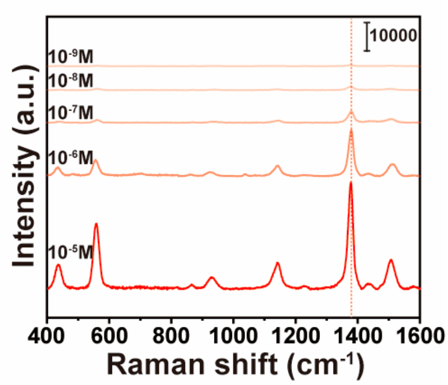

Figure S7. SERS spectrum of Tetramethylthiuram Disulfide (TMTD) acquired on the silver film substrate.
